# Sociodemographic inequalities in breast cancer screening attendance in Germany following the implementation of an Organized Screening Program: Scoping Review

**DOI:** 10.1186/s12889-024-19673-6

**Published:** 2024-08-14

**Authors:** Núria Pedrós Barnils, Victoria Härtling, Himal Singh, Benjamin Schüz

**Affiliations:** Institute for Public Health and Nursing Research, University of Bremen, Bremen, Germany

**Keywords:** Breast cancer screening, Organized screening program, Germany, Scoping review, Health inequalities

## Abstract

**Background:**

Organized breast cancer screening (BCS) programs are effective measures among women aged 50–69 for preventing the sixth cause of death in Germany. Although the implementation of the national screening program started in 2005, participation rates have not yet reached EU standards. It is unclear which and how sociodemographic factors are related to BCS attendance. This scoping review aims to identify sociodemographic inequalities in BCS attendance among 50-69-year-old women following the implementation of the Organized Screening Program in Germany.

**Methods:**

Following PRISMA guidelines, we searched the Web of Science, Scopus, MEDLINE, PsycINFO, and CINAHL following the PCC (Population, Concept and Context) criteria. We included primary studies with a quantitative study design and reviews examining BCS attendance among women aged 50–69 with data from 2005 onwards in Germany. Harvest plots depicting effect size direction for the different identified sociodemographic inequalities and last two years or less BCS attendance and lifetime BCS attendance were developed.

**Results:**

We screened 476 titles and abstracts and 33 full texts. In total, 27 records were analysed, 14 were national reports, and 13 peer-reviewed articles. Eight sociodemographic variables were identified and summarised in harvest plots: age, education, income, migration status, type of district, employment status, partnership cohabitation and health insurance. Older women with lower incomes and migration backgrounds who live in rural areas and lack private insurance respond more favourably to BCS invitations. However, from a lifetime perspective, these associations only hold for migration background, are reversed for income and urban residency, and are complemented by partner cohabitation. Finally, women living in the former East German states of Saxony, Mecklenburg-Western Pomerania, Saxony-Anhalt, and Thuringia, as well as in the former West German state of Lower Saxony, showed higher BCS attendance rates in the last two years.

**Conclusion:**

High-quality research is needed to identify women at higher risk of not attending BCS in Germany to address the existing research’s high heterogeneity, particularly since the overall attendance rate still falls below European standards.

**Protocol registration:**

https://osf.io/x79tq/.

**Supplementary Information:**

The online version contains supplementary material available at 10.1186/s12889-024-19673-6.

## Background

Breast cancer is currently the fifth leading cause of death among women in Germany, with 18,900 deaths in 2022 [1]. Socioeconomic inequalities in breast cancer (BC) survival are recognised in Europe [2] and in Germany [3]. In a phenomenon known as the “breast cancer paradox”, women with lower socioeconomic status experience higher mortality rates compared to women with higher socioeconomic status, despite lower incidence in this group [4]. Several explanations have been proposed for this paradox. Higher incidence among women with high socioeconomic status women is related to nulliparity [5], having children at an older age [6], the use of oral combined contraceptives [7], and higher screening attendance [8]. On the other hand, higher mortality and case fatality rates in women from lower socioeconomic backgrounds are linked to unhealthier lifestyles (e.g., smoking, diet) [9] and reduced screening attendance [10]. The same pattern was found between Black and Asian minority ethnic women and Caucasian women in the US [11]. Lower screening attendance leads to delayed initiation of treatment and the manifestation of more advanced tumours.

In 2003, the European Commission (EC) requested Member States to implement Organized Screening Programs (OSP), which would systematically invite women aged 50 to 69 years for bi-annual breast cancer screening (BCS), ensuring equitable access for all [12]. Germany started implementing OSP in 2005, reaching full country-wide implementation in 2009. The program is coordinated by the *Kooperationsgemeinschaft Mammographie* (Mammography Cooperation Group), with 14 regional invitation centres and 94 screening units. Registered targeted women who did not explicitly objected to be invited to screenings are bi-annually invited to take a mammography at no cost at their reference screening unit. Every screening unit, in sparsely populated areas mobile screening unit, covers an area with 800,000 to 1,000,000 inhabitants, and is headed by one or two coordinating doctors. Diagnosed breast cancers are then treated in certified breast centres [13]. Attendance rates have increased since implementation from 49 to 57% among targeted women since but are still well below the 70% EU recommendation [14]. Nevertheless, and possibly overshadowing OSP participation rates, it is important to consider the existence of grey screening in the country. This refers to mammography conducted outside the national program (i.e., via self-invitation) and thus not registered by the screening units, leading to an underestimation of the number of women undergoing screening [15].

Attendance to BCS is not uniform among eligible populations in Germany [16] or worldwide [10]. Several characteristics have been associated with BCS attendance, such as sociodemographic factors, health behaviours, health status, accessibility and logistics, attitudes, knowledge, and beliefs [17–19]. Several studies have investigated the presence of BCS sociodemographic inequalities in Germany. Missinne (2015) found a positive correlation between self-reported BCS attendance and income but no association with education [20]. Also, Heinig (2023) found no significant correlation between education and BCS attendance based on data from health insurances (claims data, onward) [15]. Regarding income differences, Lemke (2015) identified high income and lower education as predictive factors for higher BCS attendance based on screening units register-based data [21].

This suggests considerable heterogeneity in the research evidence concerning sociodemographic inequalities, and a review of socioeconomic correlates of BCS attendance in Germany is lacking. As such, a scoping review that encompasses this heterogeneity and summarises research evidence available on sociodemographic inequalities on BCS attendance in the country since the implementation of OSP could facilitate a comprehensive picture and lay the foundation for evidence-based screening interventions to increase screening participation in eligible women. The present scoping review, therefore, aims to identify sociodemographic inequalities in BCS attendance among women aged 50–69 years since the OSP implementation by answering the following questions:

1) What are the existing sociodemographic inequalities in breast cancer screening participation among targeted women following the implementation of an Organized Screening Program in Germany?

2) What are the effect sizes of the sociodemographic inequalities on breast cancer screening attendance among targeted women following the implementation of an Organized Screening Program in Germany?

## Methods

We conducted the scoping review in line with the PRISMA-ScR guidelines for scoping reviews [22], and following the five-steps methodological framework proposed by Arksey & O’Malley’s in 2005 [23]. The review protocol, including the search strategy, was registered at the Centre for Open Science (OSF) [24] and is provided as Supplementary File 1.

### Eligibility criteria

Following the recommendation for scoping reviews, we employed the PCC (Population, Concept and Context) criteria, where BCS is the concept, Germany is the context and women aged 50–69 years old are the population, as they represent the eligible screening group.

At the screening stage, studies with qualitative designs or non-primary studies, studies reporting data earlier than 2005 (i.e., date for OSP implementation) or not focused on breast cancer screening participation (e.g., focus on the program’s effectiveness), and studies that did not report information on sociodemographic variables (e.g., only general population screening attendance) were excluded. Finally, only studies published in English or German were included.

### Information sources and literature search

On January 26, 2024, the following bibliographic databases were searched for the period from January 2005 to January 2024: Web of Science, Scopus, MEDLINE (via PubMed), PsycINFO (via Ovid), and CINAHL (via EBSCO).

The search terms, developed iteratively by the research team, included descriptors of BCS, such as “mammography” or “breast cancer screening”, combined with descriptors of Germany “Germany” and the time frame 2005 onwards. All search strategies are provided as a supplementary file (Supplementary File 2). For those included articles, backward snowballing was performed using the guidelines for snowballing [29]. Furthermore, a manual search of the reference lists of the included systematic or scoping reviews was performed by one researcher (NPB) to identify further relevant articles. To identify grey literature relevant to the scoping review, two team members (NPB and VH) conducted independent searches on the websites of pertinent national public health institutions (e.g., *Bundesgesundheitsblatt*,* Bericht zum Krebsgeschehen in Deutschland*, etc.,).

### Selection of the sources of evidence

After performing the systematic search of all electronic databases, articles were retrieved, duplicates were removed, and references were imported in Rayyan [25]. Two authors, NPB and VH, independently screened the titles and abstracts and later the full texts of the studies in the next stage. Discrepancies between the researchers were discussed until a consensus was reached.

### Data items and data charting process

The data from the eligible studies were extracted in an Excel sheet that was developed, calibrated, tested and refined a priori before three researchers (NPB, VH and HS) charted the data independently. Discrepancies that arose were resolved through discussion. We charted bibliographic information (first author, year of publication, type of publication, study title, setting, aim of the study, funding sources/conflict of interest), methods (type of study, sample size of the analysis, methods of analysis, period coverage, method of reporting data, type of screening), and results (attendance rates, sociodemographic variables, other reported exposure variables, effect sizes, direction of effect sizes, p-values). When articles developed univariate and multivariate models, information from relevant variables was extracted from univariate models for further data synthesis.

### Critical Appraisal of the individual sources of evidence

The included studies were critically appraised using the National Institutes of Health (NIH) Quality Assessment Tool [26]. Two team members (NPB and VH) assessed the included studies independently, and discrepancies were discussed until a consensus was achieved.

### Synthesis of the results

Results were synthesised depending on available information. Effect sizes of sociodemographic variables were extracted from 13 peer-reviewed articles. Given the heterogeneity of study designs, we used harvest plots to summarise the data. These plots are agnostic to the outcomes and measures used and are flexible enough to include any dimension considered relevant (e.g. sample size, study design, etc.) [27]. Sociodemographic information presented in the 14 national reports was synthesised narratively. All analyses were performed with R (version 4.2.3).

## Results

**Fig. 1:**
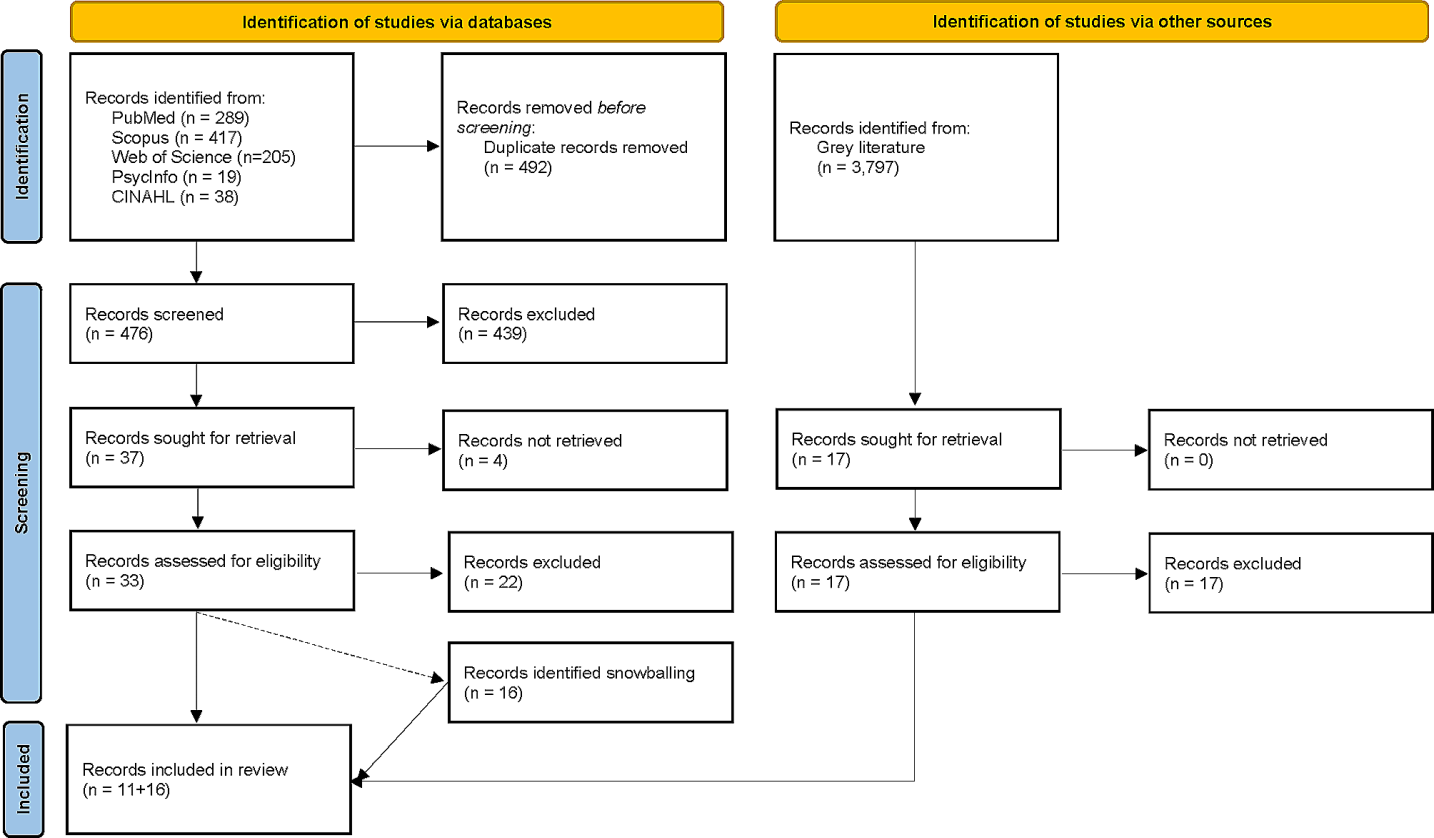
PRISMA flow chart of the search process

From the 476 titles and abstracts screened, 33 articles were included in the full-text screening. Eleven articles met the eligibility criteria and were included in the scoping review. Reasons for excluding full-text articles were not reporting breast screening attendance (*n* = 10), information on sociodemographic variables missing (*n* = 6), study before 2005 (*n* = 2), outside Germany (*n* = 1), and no quantitative or review design (*n* = 1). Two systematic reviews were identified during the full-text screening, and their reference list was checked for potential articles [4, 28]. Also, backward snowballing from included articles identified 16 further relevant records (14 national reports and 2 articles) bringing the total number of included records to 27 (Fig. 1). Grey literature identified 3,797 relevant records, from which none were finally added to the final review (details in Supplementary File 3).

### Study characteristics

The scoping review included 27 records, 13 peer-reviewed articles and 14 national reports. Supplementary File 4 reports the characteristics and summary of findings of the 27 sources. Most of the records (*n* = 20, [13–16, 20, 29–43]) utilised nationwide or nationally representative data, while seven records drew upon data from specific regions within Germany (*n* = 7, [21, 44–49]). Publication dates span from 2009 to 2023, with the highest concentration of published articles falling within the 2013–2016 timeframe (*n* = 10, [13, 20, 31–34, 41, 43, 45, 46]), closely followed by 2020–2023 (*n* = 8, [14, 15, 21, 38–40, 48, 49]). The reported data covered a broader period, ranging from 2005 to 2021. Specifically, three articles (*n* = 3, [20, 42, 47]) and two national reports (*n* = 2, [29, 30]) pertain to the OSP implementation phase spanning 2005–2009. The remaining records (*n* = 22, [13–16, 21, 31–41, 43–46, 48, 49]) report data from 2009 onwards, when the OSP was fully implemented. Four included articles (*n* = 4, [15, 21, 44, 45]) had a cohort study design, nine articles (*n* = 9 [16, 20, 41–43, 46–49]), and all national reports (*n* = 14, [13, 14, 29–40]) had a cross-sectional design. The sample sizes of the records ranged from *n* = 237 [42] to *n* = 1,151,000 [44] for peer-reviewed articles and *n* = 4,864,574 [31] to *n* = 5,887,028 for national reports [14]. Peer-reviewed articles reported three methods for collecting data: self-reported data (*n* = 7, [16, 20, 21, 42, 43, 47, 49]), claims data (*n* = 2, [15, 41]), screening units register-based data (*n* = 3, [45, 46, 48]). One article used claims and register-based data (*n* = 1, [44]). All national reports reported screening units register-based data (*n* = 14, [13, 14, 29–40]).

Three articles reported BCS attendance for the last year (*n* = 3, [41, 42, 44]), five reported attendance in the last two years (*n* = 5, [16, 43, 45–47]) and five reported never having attended to BCS (*n* = 5, [15, 20, 21, 48, 49]). All national reports reported attendance in the last two years.

### Critical appraisal of included sources of evidence

**Fig. 2:**
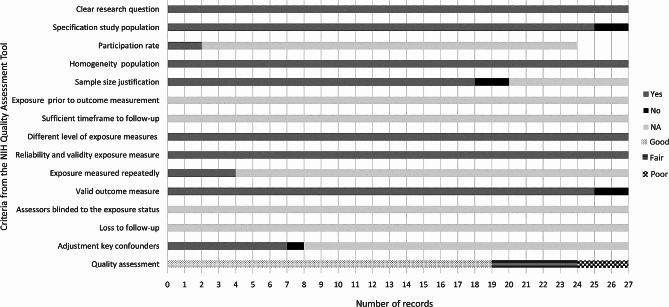
Critical Appraisal of 27 included records (NIH Quality Assessment Tool for Observational Cohort and Cross-Sectional Studies)

The methodological quality of all included records was rated following the NIH Quality Assessment Tool for Observational Cohort and Cross-Sectional Studies given its suitability for the research designs of the included records [26]. All 14 national reports were rated as good, five peer-reviewed articles were rated as good (*n* = 5, [15, 21, 43, 45, 46]), five as fair (*n* = 5, [16, 20, 41, 42, 44]) and three as poor (*n* = 3, [47–49]). A study was considered to be good if all the applicable criteria were met, fair if one was not met and poor if two or more were not met. The overall rating of different aspects considered is represented in Fig. 2, and the reasons behind each record’s rating are in Supplementary File 5.

### Breast cancer screening attendance rates across studies

Cross-sectional studies reported an average attendance rate of 55.10% (SD = 9.85), cohort studies reported 74.67% (SD = 17.94). The average attendance rate of all records was 57.42% (SD = 12.40). Average lifetime attendance rate was 72.54% (SD = 16.32), attendance in last two years was 53.83% (SD = 8.15), attendance in the last year was 52.3% (SD = 8.34). Two articles did not report overall BCS attendance rates (*n* = 2, [41, 45]).

Studies with self-reported attendance reported an average attendance rate of 63.84% (SD = 20.27), those based on claims and screening units register-based data showed an average attendance of 54.97% (SD = 6.99).

OSP implementation phase (2005–2009) and the full OSP implementation (2009 onwards) also showed different participation rates: 47.94% (SD = 6.87) and 59.19% (SD = 12.47), respectively. There was an increase in the lifetime attendance rate from 72.54% (SD = 16.32) to 78.48% (SD = 10.22) (*n* = 4, [15, 21, 48, 49]) when only assessing studies carried since the full OSP implementation.

### Evaluation reports of the German mammography screening programme

Fourteen of the included sources are national evaluation reports of the German Mammography Screening Programme, initiated in 2005 and conducted by the *Kooperationsgemeinschaft Mammographie* [50]. The reports graphically presented participation rates per federal state based on the screening units register-based data, and, as such, have the potential to detect regional inequalities in attendance. No federal state has yet reached the EU recommended rate of > 70% participation of the target population. Still, Lower Saxony, Saxony, Mecklenburg-Western Pomerania, Saxony-Anhalt and Thuringia have had the highest participation rates over the last five years [14].

### Sociodemographic inequalities in breast cancer screening participation

**Fig. 3:**
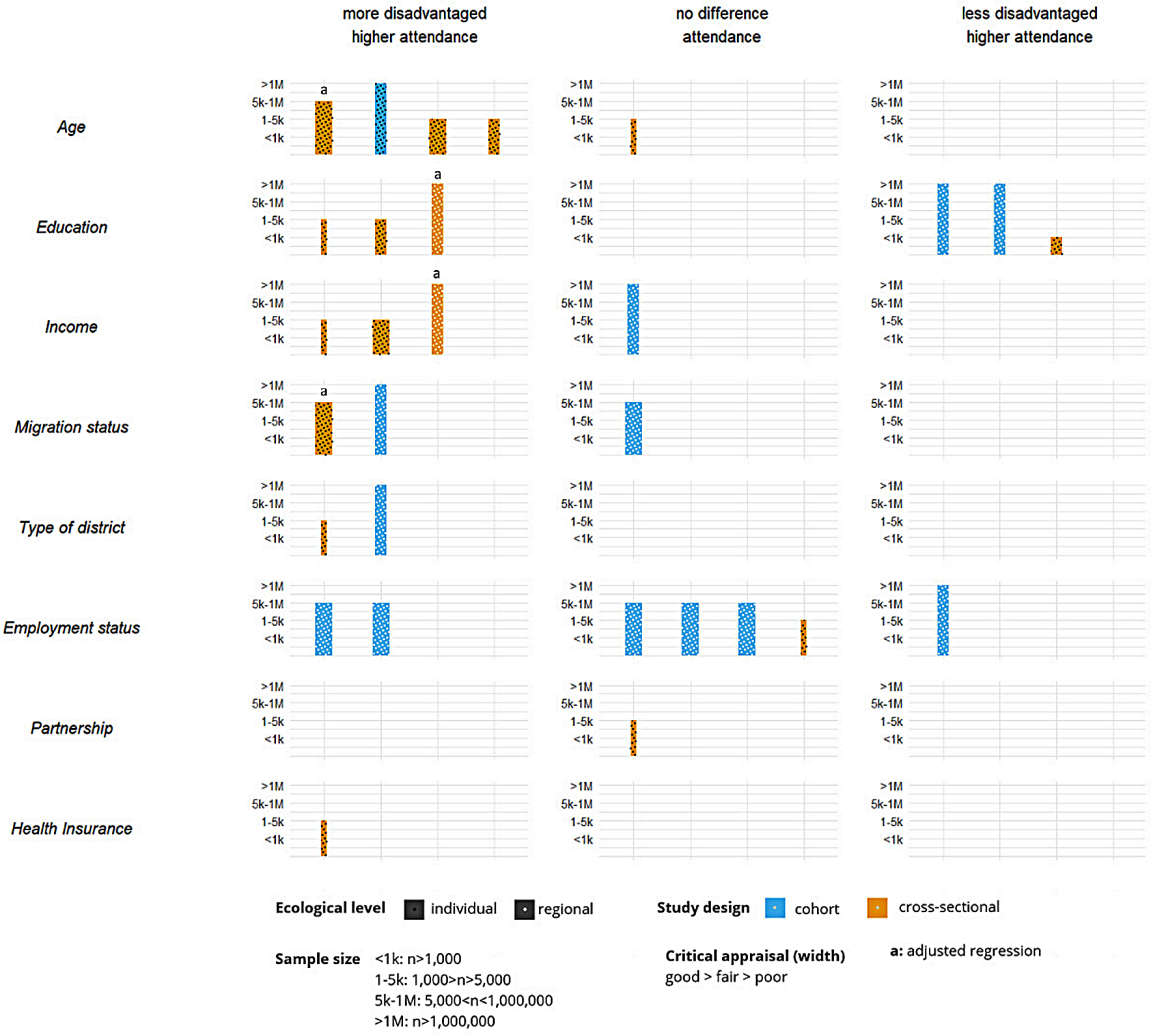
Harvest plot displaying correlations between sociodemographic variables and last one-two years BCS attendance in Germany

**Fig. 4:**
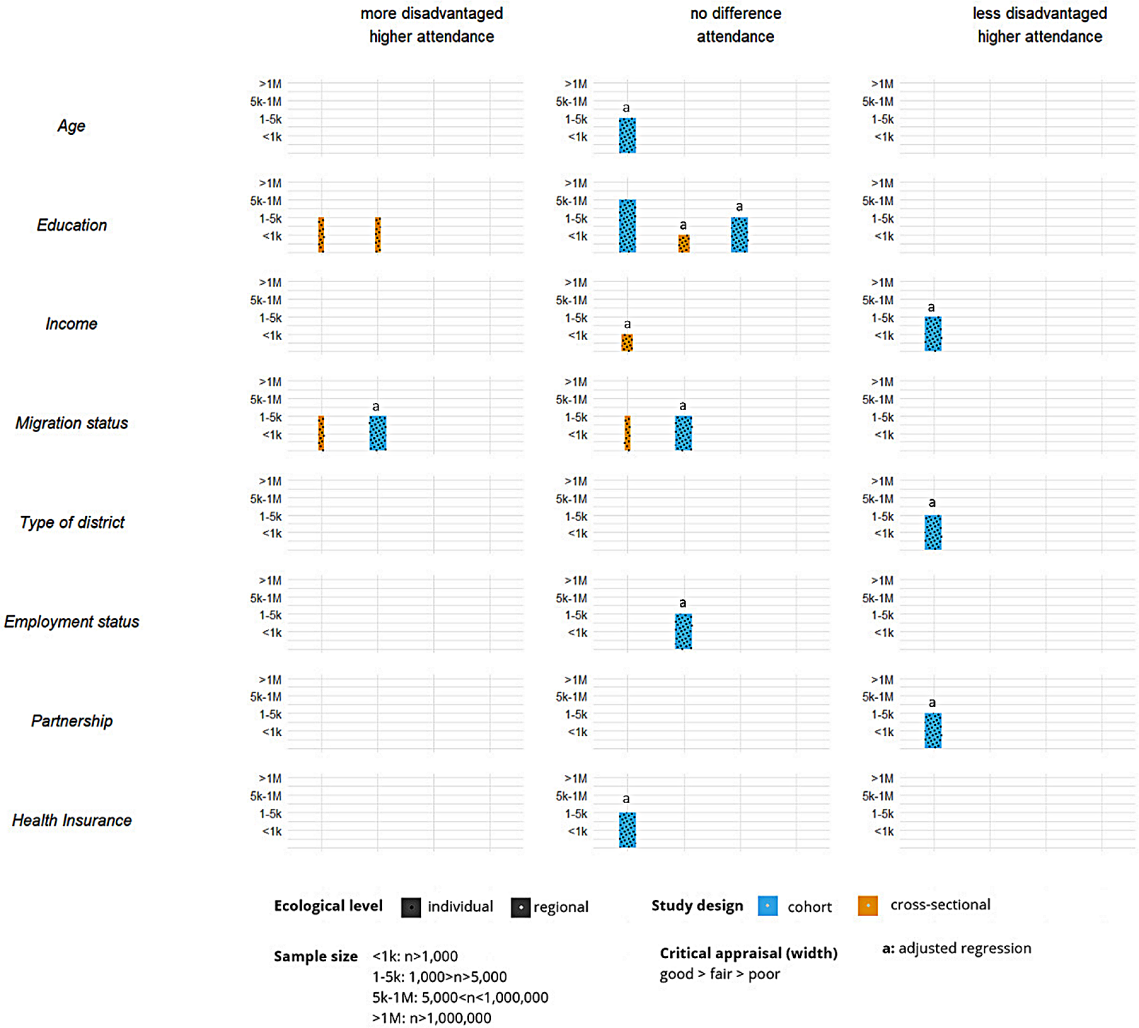
Harvest plot displaying correlations between sociodemographic variables and lifetime BCS attendance in Germany

Sociodemographic inequalities in BCS participation were examined by harvest plots based on vote counting for sociodemographic variables reported in at least two articles: age, education, income, migration status, type of district, employment status, partnership cohabitation and health insurance. Two harvest plots were plotted for the distinct reported outcomes: Attendance during the last one or two years (Fig. 3) and lifetime BCS attendance (Fig. 4). Details on the harvest plots can be found in Supplementary File 6.

Each bar represents a study and illustrates five characteristics of the study: the height of the bars indicates the sample size of the study, the width of the bars reflects the study quality based on the NIH critical appraisal tool, colour denotes the study type (blue: cohort; yellow: cross-sectional), and filling pattern indicates sociodemographic data level source (black: individual; white: regional).

### Age (6 effects)

Among those reporting attendance in the last one/two years, 4/5 suggested higher attendance in older women [16, 43, 44, 46], and one suggested no correlation [47]. One study found no significant relationship between age and lifetime BCS attendance [21].

### Education (11 effects)

Two studies (three effects) suggested higher attendance in the last one/two years with higher education [42, 44], and three studies suggested the opposite [16, 41, 47]. Two studies revealed a negative correlation [48, 49], and three studies found no relationship [15, 20, 21] for lifetime BCS attendance.

### Income (4 effects)

Three studies found a negative correlation between income and attendance in the last one/two years [41, 43, 47], and one study found no correlation [44]. One study found no correlation between lifetime BCS [20], and one study found a positive correlation [21].

### Migration status (7 effects)

4/7 studies identified higher lifetime BCS attendance in those with migration status (2 lifetime [21, 49]; 2 last two years [44, 46]). Three studies found no correlation [21, 45, 48]. Here, two effects from the same study illustrated a positive correlation between first-generation migrants and locals, but there was no correlation when estimating second-generation migrants versus locals [21].

### Type of district (3 effects)

Two studies found that living in a rural area favours last one/two years BCS attendance (*n* = 2, [44, 47]), whereas one found no correlation for lifetime BCS attendance (*n* = 1, [21]).

### Employment status (7 effects)

Lemke (2015) contributed 5/7 effects, showing a contextual negative association in two and no relationship in three cases [45]. Two more studies also found no relationship with BCS attendance [21, 47]. Conversely, one study identified a positive correlation between employment status and BCS attendance in the last year [44].

### Partnership cohabitation (2 effects)

One study found a positive relationship between cohabitating and lifetime BCS [21], and another found no relationship for BCS in the last two years [47].

### Health insurance (2 effects)

One study observed no correlation between health insurance and lifetime BCS [21]. Still, another study identified that compulsory insurance (as opposed to private insurance) was associated with BCS in the last two years [47].

## Discussion

This scoping review aimed to identify sociodemographic inequalities in BCS attendance in Germany following the implementation of the OSP. Eight sociodemographic variables were identified: age, education, income, migration status, type of district, federal state, employment status, partnership cohabitation, and health insurance.

Women are more likely to attend BCS following invitation letters within two years or less as they age, have lower incomes, have a migration background, reside in rural areas, live in certain federal states such as Lower Saxony, Saxony, Mecklenburg-Western Pomerania, Saxony-Anhalt, and Thuringia, and are insured within the compulsory health insurance system (i.e., do not hold private health insurance; based on one study only). Lifetime BCS attendance is more likely in women with higher incomes, migration backgrounds, urban residency (based on one study only), and cohabitating status with a partner (based on one study only).

Our finding regarding age is similar to a scoping review in Spain [51]. However, the lifetime impact on overall BCS attendance requires further investigation, as in our scoping review, there is a substantial imbalance in research outputs- only one study focused on lifetime attendance [21].

The size and direction of the impact of educational attainment on attending BCS within two years or less remains unclear, as we identified studies reporting opposing results. Considering the quality of the assessed studies, those suggesting higher education as a protective factor yielded higher quality. In this line, Damiani’s (2015) international systematic review found that highly educated women were the most likely to undergo BCS after invitation [52]. Studies examining lifetime BCS attendance found different results, showing that in over half of the studies with high quality, there was no correlation, and in less than half with lower quality, there was a negative correlation between educational attainment and BCS attendance. There were also heterogeneous results concerning the association between income and BCS attendance. Lower income women depicted higher short-term BCS attendance rates and higher income women higher long-term BCS attendance rates.

These findings challenge the breast cancer screening paradox whereby women with higher socioeconomic status are more likely to engage in BCS. In our scoping review, there was no straightforward relationship identified between higher educational attainment or income and higher participation rates. A comparable pattern was identified by a recent international systematic review [10], which indicated that women with higher levels of education or income were no more likely to attend BCS than those with intermediate levels of education or income. It is hypothesised that high SES women utilise alternative screening services, such as grey screening, to a greater extent, and could also have larger concerns about the overall benefits of screenings [53]. Indeed, Berens (2015) exposed that women with higher educational attainment were more likely to make informed choices than those with lower educational attainments in Germany [54].

Moreover, in the context of Germany, a considerable proportion of women with high income may have a private health insurance, often as a result of their employment status as civil servants [55]. For those citizens with private health insurance, the utilisation of any preventive service can be initially borne by the individual and subsequently reimbursed, unless the overall out-of-pocket expenditure in a given year does not exceed a specified threshold (e.g., 500€), potentially jeopardising the use of these services [56]. Indeed, in our review one study suggests that holding private health insurance is associated with lower BCS attendance in the last two years . In line with this finding , private health insurance in Spain also negatively correlates with last two years BCS attendance [57].

Women with migrant backgrounds appear more likely to attend BCS in the short and long term. Over half the studies with fair to good quality support this trend. Contrarily, several studies assessing BCS attendance in other high-income countries [58–60] or assessing general cancer screening attendance in Germany [61, 62] found the inverse tendency.

Furthermore, attending BCS within the last two years or less was positively correlated with living in rural areas. Analogously, Serral (2018) found the same association [57]. In the reviewed studies, BCS attendance was higher in Lower Saxony, Saxony, Mecklenburg-Western Pomerania, Saxony-Anhalt, and Thuringia. Except for Lower Saxony, all these states conform are former East German states. Großmann (2023) also noted higher BCS attendance rates in former East German states and suggested that the former centralised healthcare and prevention systems, perceived as state tasks, contributed to women viewing participation as a social responsibility [63].

About two-thirds of the included records found no significant difference between regions with higher and lower unemployment levels, while the remaining third suggested a slightly higher short term BCS attendance in regions with higher unemployment levels. These results are in accordance with Serral (2016), who showed, after adjusting by age, a positive relationship between not working and BCS attendance in Spain [57], while Jensen (2012) found the opposite relationship in Denmark [64].

Partnership cohabitation was positively associated with lifetime BCS but not with last two years or less BCS attendance. The study with a positive relation had a more robust design and better-quality assessment. Similarly, Jolidon (2022) found significantly higher attendance rates among married women compared to unmarried women in Switzerland [65]. Lastly, no study included women with disabilities in their analysis. Andiwijaya’s (2022) systematic review revealed that having a disability was negatively associated with BCS attendance [66].

### Strengths and limitations

To our knowledge this is the first scoping review to identify sociodemographic inequalities in BCS attendance among women aged 50–69 years since the implementation of the OSP in Germany. In accordance with the PRISMA-ScR guidelines, the search and the screening process was conducted in a transparent and reproducible manner, and the results were visually summarised using harvest plots.

The scoping review identified heterogeneous study designs, which corresponded with the mixed findings: cross-sectional studies indicated nearly 20% lower participation rates than cohort studies. Similarly, there was almost a 20% difference between the average participation rates over the past one or two years and lifetime participation rates (i.e., being lifetime participation higher). Slightly minor differences were found with the data collection method. Here, claims or screening units register-based data reported a 9% higher prevalence than self-reported participation. Finally, BCS participation prevalence during the OSP implementation phase (2005–2009) was 12% lower than after total implementation, and was reported as a compound of formal OSP participation and self-invited participation (i.e., grey screening) [29, 30, 47]. The varied BCS prevalence across study characteristics might align with our scoping review’s mixed findings in sociodemographic inequalities.

Lastly, given the heterogeneity of study designs, it is impossible to distinguish correlation from causation. Accordingly, more cohort studies with objective data sources, such as claims and screening units register-based data, are needed.

## Conclusions and implications

This scoping review shows considerable heterogeneity in sociodemographic inequalities in BCS attendance following the implementation of the OSP in Germany. Older women, women with lower incomes, women with migration background, women living in rural areas, women living in former East Germany states and women who are insured in the statutory insurance system respond more favourably to BCS invitations. Regarding lifetime BCS attendance, these associations only hold for migration background, are reversed for income and urban residency, and are complemented by partner cohabitation.

Given that overall attendance is well below European standards, specific sociodemographic groups should be targeted in BCS participation campaigns. At the same time, more high-quality research is needed to identify women at higher risk of not attending BCS in Germany to provide better evidence.

### Electronic supplementary material

Below is the link to the electronic supplementary material.

Supplementary Material 1

Supplementary Material 2

Supplementary Material 3

Supplementary Material 4

Supplementary Material 5

Supplementary Material 6

## Data Availability

All data generated or analysed during this study are included in this published article and its supplementary files.
