# Supplementary material for: Sociodemographic inequalities in breast cancer screening attendance in Germany following the implementation of an Organized Screening Program: Scoping Review
Source: BMC Public Health. 2024 Aug 14;24:2211. doi: 10.1186/s12889-024-19673-6 (PMC11323608; doi:10.1186/s12889-024-19673-6)
Supplement: Supplementary file 1 — Supplementary Material 1 [file 12889_2024_19673_MOESM1_ESM.docx]

**PROTOCOL FOR A SCOPING REVIEW**

Table of Contents

[General information 2](#_Toc157517625)

[Title 2](#_Toc157517626)

[Research team and organizational affiliations 2](#_Toc157517627)

[Timeline 2](#_Toc157517628)

[Funding sources 2](#_Toc157517629)

[Study details according to the PRISMA-ScR checklist 2](#_Toc157517630)

[Item 1. Title 2](#_Toc157517631)

[Item 2. Abstract 2](#_Toc157517632)

[Item 3. Rationale 2](#_Toc157517633)

[Item 4. Objectives 3](#_Toc157517634)

[Item 5. Protocol and registration 3](#_Toc157517635)

[Item 6. Eligibility criteria 3](#_Toc157517636)

[Item 7. Information sources 4](#_Toc157517637)

[Item 8. Search 4](#_Toc157517638)

[Item 9. Selection of sources and evidence 4](#_Toc157517639)

[Item 10. Data charting process 4](#_Toc157517640)

[Item 11. Data items 5](#_Toc157517641)

[Item 12. Critical appraisal of individual sources of evidence 5](#_Toc157517642)

[Item 13. Summary measures 5](#_Toc157517643)

[Item 14. Synthesis of the results 5](#_Toc157517644)

[Item 15. Risk of bias across studies 5](#_Toc157517645)

[Item 16. Additional analyses 5](#_Toc157517646)

[Items 17-26: Results / Discussion 5](#_Toc157517647)

[Item 27: Funding 5](#_Toc157517648)

[References 6](#_Toc157517649)

# General information

## Title

A scoping review of sociodemographic inequalities on the uptake of breast cancer screening among targeted women in Germany since the implementation of the Organized Screening Program

## Research team and organizational affiliations

Núria Pedrós Barnils^1^, Victoria Härtling^1^, Himal Singh^1^, Ulrike Haug^1,2^, Benjamin Schüz^1^

1. Institute for Public Health and Nursing Research, University of Bremen, Bremen, Germany

2. Department of Clinical Epidemiology, Leibniz Institute for Prevention Research and Epidemiology

## Timeline

1. Start date: 26.01.2024

2. Anticipated completion date: 30.06.2024

3. Project stage at registration (30.01.2024):

• The final search has been run.

## Funding sources

There are no sources of financial support for the present scoping review.

# Study details according to the PRISMA-ScR checklist

## Item 1. Title

A scoping review of sociodemographic inequalities on the uptake of breast cancer screening among targeted women in Germany since the implementation of the Organized Screening Program

## Item 2. Abstract

Not applicable at the protocol stage

## Item 3. Rationale

According to the last available statistics of the German Federal Statistical Office (Statistisches Bundesamt), malignant neoplasm of the breast was the fifth leading cause of death in Germany in 2020, with 18,500 deaths (1). In 2003, the European Commission urged Member States to establish preventive organized screening programs (OSP) that bi-annually invite women aged 50 to 69 years for breast cancer screening (BCS) (2). Germany started implementing OSP in 2005, and the participation rate (n° participants/n° invited) has since fluctuated. In 2021, the participation rate among all women who received an invitation was of 51%. Moreover, among federal states, the participation rates vary from 43% to 59%, with Lower Saxony, Saxony, and Mecklenburg-Pomerania having the highest rates (3).

Several studies investigated which subgroup of targeted women have never attended to BCS in Germany. Nationally and within the scheme of OSP, Missinne et al., using the Survey on Health, Aging and Retirement database of 2008/2009 in a cross-sectional study design, found a positive significant association between BCS attendance and income adjusted by age, whereas no significant association was found for education (4). Moreover, in a longitudinal study, Heinig et al. also found no significant association with education in the large German Pharmacoepidemiologic Research Database based on claims data (5). Nevertheless, in a longitudinal study in the Rhine-Main region, higher income and lower educational status were found to be significantly associated with higher participation rates in mammography, adjusted by age (6). In another longitudinal study in the city of Dortmund migrant status (based on nationality) and long-term unemployment were statistically associated with lower mammography attendance (7). Some studies have raised concerns about the existence of gray screening (i.e., mammography taking place outside OSP) and its potential overshadowing of participation rates in OSP (5).

All these results have not been systematically reviewed to date. A scoping review of all studies conducted in the country since the implementation of OSP can facilitate a broad overview on the sociodemographic variables are most often assessed for researching BCS attendance inequalities. Identifying the size, direction, and magnitude of these inequalities, along different studies design, and presenting the results in a structured and comprehensive manner can additionally inform health policy planning in the future.

## Item 4. Objectives

The scoping review aims at identifying sociodemographic inequalities in the uptake of breast cancer screening among targeted women since the implementation of the Organized Screening Program in Germany as well as assessing effect sizes of these sociodemographic dimensions. The review questions are:

1) What are the existing sociodemographic inequalities in the uptake of breast cancer screening among targeted women since the implementation of the Organized Screening Program in Germany?

2) What are the effect sizes of the sociodemographic inequalities on the uptake of breast cancer screening among targeted women since the implementation of the Organized Screening Program in Germany?

## Item 5. Protocol and registration

The protocol has not been previously registered elsewhere.

## Item 6. Eligibility criteria

The eligibility criteria for this scoping review are based on the PCC (Population, Concept, and Context) criteria recommended for scoping reviews and listed in Table 1.

**Table 1.** Inclusion and Exclusion criteria for the scoping review.

| Inclusion criteria | Exclusion criteria |
| --- | --- |
| English or German language | Non-English or German language |
| Studies covering data from 2005 onwards | Studies only covering data earlier than 2005 |
| Studies published from 2005 onwards | Studies published before 2005 |
| Studies focused on breast cancer screening attendance in Germany (Concept, Context) | Studies on breast cancer screening attendance outside of Germany  Studies focusing on the benefits and harms of breast screening  Studies focusing on screening in the context of inherited breast cancer |
| Studies targeting women aged 50-69 years old (Population) | Studies that do not report results for the relevant age group 50-69 |
| Studies that considered the uptake of formal or gray breast cancer screening |  |
| Study design: descriptive quantitative design such as cross-sectional studies or cohort studies and reviews | Study design: qualitative designs |
| Access to full-text | Not access to full-text |
| Publication types: a) journal articles, b) health reports | Other publication types: comment, correction, letter, editorial, protocol, oral presentation, or posters |
| Publication status: published | Publication status: pre-print |

## Item 7. Information sources

The following bibliographic databases will be searched from 2005 to January 2024: Web of Science, Scopus, MEDLINE (via PubMed), PsycINFO (via Ovid), and CINAHL (via EBSCO).

To identify grey literature, a search for health reports relevant to this review will be conducted on the websites of appropriate national institutions within the same time frame.

## Item 8. Search

The search will be conducted in English by one researcher. We aim to include sources in English or German. The search syntax in the bibliographic databases will include the following basic keywords, specific search fields, and Boolean operators. The search syntax will include synonyms and the most relevant subject terms reflecting the PCC criteria relevant for this scoping review:

**Concept** (Breast Cancer Screening) *AND* **Context** (Germany)

To be broader and make sure no other wording was used for attendance/participation, we have not added this word to the search strategy. Moreover, the **Population** (P – women aged 50-69) will not be included in the search strategy since they might not be implicit in the title – abstract and, therefore, not explicitly written. They are applied in the exclusion criteria.

## Item 9. Selection of sources and evidence

The electronic search results will be uploaded to the online scoping review management software Rayyan, which will be used throughout the review process. Initially, the deduplication process will take place. Following, two researchers will independently evaluate the eligibility of titles and abstracts in the first step. Subsequently, two researchers will independently acquire and review the full texts of potentially relevant records. Any discrepancies between the researchers during the title and abstract screening and the full-text review stages will be addressed through discussion until a consensus is achieved. If no consensus is reached, a third researcher will be involved to make the final decision.

Moreover, grey literature and health reports on breast cancer screening in Germany will be manually searched and included when relevant.

## Item 10. Data charting process

A data coding sheet will be developed a priori in Excel. General information will be extracted from the included texts (e.g., bibliographic information, methods, and research results). One researcher will perform data coding. For quality assurance, a random sample of 8 included texts will be coded by another researcher as pilot testing for the coding process. The data coding sheet will be revised in an iterative process during the pilot coding and calibrated within the team. In case of existing missing data, the reviewer team will contact the authors and ask for the unreported data.

## Item 11. Data items

The following data items will be extracted from the included texts:

1) Bibliographic information (first author, year of publication, study title, setting, aim of the study, funding sources/conflict of interest)

2) Methods (type of study, sample size of the analysis, methods of analysis, period coverage, method of reporting data)

3) Research results (a measure used to describe participation, sociodemographic variables, other reported exposure variables, effect sizes, the direction of the effect size, p-value of the effect size, significance of the effect size)

## Item 12. Critical appraisal of individual sources of evidence

A critical appraisal of the included studies will be conducted. Since three different study designs are included (cross-sectional, and cohort and reviews), different tools are used. Both for cross-sectional and cohort studies, STROBE checklists will be used (8). STROBE checklists aim at strengthening the reporting of observational studies in epidemiology in the three main analytical designs used in observational research: cohort, case-control and cross-sectional studies.

If systematic reviews are identified, the CASP checklist will be applied (9). The foundational CASP checklists (randomized controlled trials and systematic reviews) were developed based on the JAMA 'Users’ guides to the medical literature 1994 and tested with healthcare practitioners. For each subsequent checklist, a panel of experts created and piloted the checklist making adjustments when necessary.

Two reviewers will critically assess the included studies independently. Any discrepancies between the researchers will be addressed through discussion until a consensus is achieved. If no consensus is reached, a third researcher will be involved to make the final decision.

## Item 13. Summary measures

*Not applicable for scoping reviews.*

## Item 14. Synthesis of the results

A narrative synthesis will descriptively summarize the sociodemographic inequalities assessed for breast cancer screening in Germany. Depending on study heterogeneity, we will quantitatively summarize the data. Depending on heterogeneity within the studies, we will rely on vote counting (if possible, with harvest plots), albatross plots, or random-effects meta-analyses.

## Item 15. Risk of bias across studies

Not applicable for scoping reviews.

## Item 16. Additional analyses

Not applicable for scoping reviews.

## Items 17-26: Results / Discussion

Not applicable at the protocol stage.

## Item 27: Funding

There are no sources of financial support for the present scoping review.

# References

1. Statistisches Bundesamt. Causes of death (2023) [online] Available at: <https://www.destatis.de/EN/Themes/Society-Environment/Health/Causes-Death/_node.html#sprg267092>. Accessed: 10.01.2024

2. European Comission. European guidelines on breast cancer screening and diagnosis (2022) [online] Available at: https://healthcare-quality.jrc.ec.europa.eu/en/ecibc/european-breast-cancer-guidelines. Accessed: 10.01.2024

3. Hand E. Jahresbericht Evaluation 2021, Deutsches Mammographie-Screening-Programm. Berlin: Kooperationsgemeinschaft Mammographie; 2023.

4. Missinne S, Bracke P. A cross-national comparative study on the influence of individual life course factors on mammography screening. Health Policy. 2015;119(6):709-19.

5. Heinig M, Schäfer W, Langner I, Zeeb H, Haug U. German mammography screening program: adherence, characteristics of (non-)participants and utilization of non-screening mammography—a longitudinal analysis. BMC Public Health. 2023;23(1).

6. Pokora RM, Büttner M, Schulz A, Schuster AK, Merzenich H, Teifke A, et al. Determinants of mammography screening participation–a cross-sectional analysis of the German population-based Gutenberg Health Study (GHS). PLOS ONE. 2022;17(10):e0275525.

7. Lemke D, Berkemeyer S, Mattauch V, Heidinger O, Pebesma E, Hense HW. Small-area spatio-temporal analyses of participation rates in the mammography screening program in the city of Dortmund (NW Germany) Biostatistics and methods. BMC Public Health. 2015;15(1).

8. STROBE Checklists. STROBE (2024). [online] Available at: <https://www.strobe-statement.org/checklists/> Accessed: Date Accessed: 30.01.2024.

9. Critical Appraisal Skills Programme (2018). CASP Systematic Review Checklist. [online] Available at: <https://casp-uk.net/checklists/casp-systematic-review-checklist-fillable.pdf>. Accessed: Date Accessed: 30.01.2024.

**PRISMA extension for scoping reviews (PRISMA-ScR) checklist from:**

Tricco AC, Lillie E, Zarin W, O'Brien KK, Colquhoun H, Levac D, et al. PRISMA extension for scoping reviews (PRISMA-ScR): checklist and explanation. Annals of Internal Medicine. 2018;169(7):467-73. doi:10.7326/M18-0850
