# Supplementary material for: Sociodemographic inequalities in breast cancer screening attendance in Germany following the implementation of an Organized Screening Program: Scoping Review
Source: BMC Public Health. 2024 Aug 14;24:2211. doi: 10.1186/s12889-024-19673-6 (PMC11323608; doi:10.1186/s12889-024-19673-6)
Supplement: Supplementary file 2 — Supplementary Material 2 [file 12889_2024_19673_MOESM2_ESM.docx]

**Supplementary File 2. Search strategy of the scoping review**

**Date of search**: 26/01/2024

**PubMed**

(("Germany"[Title/Abstract] OR "Germany"[MeSH Terms]) AND ("breast cancer screening"[Title/Abstract] OR "Mammography"[Title/Abstract] OR "Mammography"[MeSH Terms] OR "ultrasonography, mammary"[MeSH Terms])) AND (2005:2024[pdat])

**Scopus**

( TITLE-ABS-KEY ( germany ) AND TITLE-ABS-KEY ( mammography OR "breast cancer screening" ) ) AND PUBYEAR > 2004 AND PUBYEAR < 2025

**Web of Science**

(TS=(Germany)) AND TS=("breast cancer screening" OR Mammography) Timespan: 2005-01-01 to 2024-01-26 (Publication Date)

**PsycINFO**

Germany.ab. or Germany.mh. Mammography.ab. or Mammography.mh. or "breast cancer screening".ab. or "breast cancer screening".mh.1 and 2 2005 - 2024

**CINAHL**

AB germany AB mammography OR AB breast cancer screening AB S1 AND S2 " Limiters - Publication Date: 20050101-20241231 Expanders - Apply equivalent subjects Search modes - Boolean/Phrase "
