# Supplementary material for: Sociodemographic inequalities in breast cancer screening attendance in Germany following the implementation of an Organized Screening Program: Scoping Review
Source: BMC Public Health. 2024 Aug 14;24:2211. doi: 10.1186/s12889-024-19673-6 (PMC11323608; doi:10.1186/s12889-024-19673-6)
Supplement: Supplementary file 3 — Supplementary Material 3 [file 12889_2024_19673_MOESM3_ESM.docx]

**Supplementary File 6.** **Harvest plots building process**

The results of the records included in the scoping review were synthesised using vote counting. Harvest plots were displayed, showing the effect size of the relationship between sociodemographic variables and BCS attendance (last two years or lifetime) favoured advantage or disadvantaged populations. For the eight included sociodemographic variables, the following categories were considered advantaged and disadvantaged:

- Age: young (advantaged), old (disadvantaged)
- Education: high (advantaged), low (disadvantaged)
- Income: high (advantaged), low (disadvantaged)
- Migration status: local (advantaged), migrant (disadvantaged)
- Type of district: urban (advantaged), rural (disadvantaged)
- Employment status: low/no (advantaged), high/yes (disadvantaged)
- Partnership: yes (advantaged), no (disadvantaged)
- Health insurance: yes (advantaged), no (disadvantaged)

There were some sociodemographic variables in which neither the most advantaged nor more disadvantaged categories presented higher participation rates but rather the middle one. That is the case for:

- Age (Berens, 2014, Starker, 2017)
- Education (Starker, 2017 and Czwikla, 2019 - the proportion of employees with an academic degree)
- Income (Starker, 2013)
- Migration background (Czwikla 2019 - the proportion of the foreign population within a district)

In these cases, the more advantaged category (younger women for age, higher education for education, higher income for income and district with low proportion of foreign population for regional form of migration) were considered advantaged, and all the remaining categories (i.e., the middle groups) were considered disadvantaged.

However, in the case of education, Czwikla, 2019 (Proportion of employees with an academic degree), the author concluded, “Quintiles 2 and 3 had higher mammography attendance than 1, quintiles 4 and 5 had lower mammography attendance than 1. Therefore, districts with a higher proportion of employees with academic degrees were positively associated with mammography attendance.” Hence, for building the harvest plot, we considered higher participation among advantaged educational groups (districts with more employees with academic degrees).

Finally, when a variable was reported as interacting with another variable (e.g., education interacting with age in Starker, 2017), the effect direction was used in the harvest plot only when the variable was not reported otherwise (e.g., in Kuehnle, 2021 effect of migration on BCS attendance was reported univariable and stratified by education) and the direction common across the different stratifications.
