# Supplementary material for: Sociodemographic inequalities in breast cancer screening attendance in Germany following the implementation of an Organized Screening Program: Scoping Review
Source: BMC Public Health. 2024 Aug 14;24:2211. doi: 10.1186/s12889-024-19673-6 (PMC11323608; doi:10.1186/s12889-024-19673-6)
Supplement: Supplementary file 5 — Supplementary Material 5 [file 12889_2024_19673_MOESM5_ESM.docx]

**Supplementary File 3. Grey literature search**

| **Side search**  **(URL)** | **Records screened** | **Full text screened** | **Included** |
| --- | --- | --- | --- |
| **Bundesgesundheitsblatt**  (https://link.springer.com/search?new-search=true&facet-journal-id=103&query=*&content-type=article&date=custom&dateFrom=2005&dateTo=2024&sortBy=relevance)   - Only 2005-2024 | 3721 | 7 | 0 |
| **Journal of Health Monitoring**  (https://www.rki.de/DE/Content/Gesundheitsmonitoring/JoHM/JoHM_alle/johm_alle_node.html) | 68 | 2 | 0 |
| **Zentrum für Krebsregisterdaten**  (https://www.rki.de/DE/Content/Gesundheitsmonitoring/Krebsregisterdaten/krebs_node.html)   - Bericht zum Krebsgeschehen in Deutschland 2016 - Krebs in Deutschland für 2007/2018 bis 2019/2020 (7) | 8 | 8 | 0 |
