# Supplementary material for: Sociodemographic inequalities in breast cancer screening attendance in Germany following the implementation of an Organized Screening Program: Scoping Review
Source: BMC Public Health. 2024 Aug 14;24:2211. doi: 10.1186/s12889-024-19673-6 (PMC11323608; doi:10.1186/s12889-024-19673-6)
Supplement: Supplementary file 6 — Supplementary Material 6 [file 12889_2024_19673_MOESM6_ESM.docx]

**Supplementary File 4. Table summary of the twenty -seven included records’ characteristics**

| Study (Author, year) | Region | Period covered | Study title | Study design ^a^ | Data collection method ^b^ | Attendance measure ^c^ | Attendance rate (%) | Sample size | Sociodemographic variables | Other variables reported |
| --- | --- | --- | --- | --- | --- | --- | --- | --- | --- | --- |
| Albert, 2012 (47) | 10 federal states in Germany | 2008 | The Population-Based Mammography Screening Programme in Germany: Uptake and First Experiences of Women in 10 Federal States | CS | S | L2 | 37.3 | 3,226 | Age  Type of district  Children  Partnership cohabitation  Education  Employment status Household income  Health insurance | BMI  Life quality  Medicament use |
| Berens, 2014 (46) | Duisburg, Bielefeld, Paderborn, Hamburg, and Berlin | 2010-2011 | Participation in breast cancer screening among women of Turkish origin in Germany – a register-based study | CS | R | L2 | 49.2 | 423,649 | Age  Migration background  (Turkish vs non-Turkish) |  |
| Czwikla, 2019 (44) | Lower Saxony | 2011-2014 | Assessing and Explaining Geographic Variations in Mammography Screening Participation and Breast Cancer Incidence | CH | C & R | L1 | 57.5 & 58.9 | 1,151,000 | Age  *Regional variables:* Unemployment rate  Average household income per inhabitant  Proportion of employees without qualification  Proportion of employees with an academic degree  Proportion of foreign population  Type of district | Elixhauser comorbidities |
| Evaluationsbericht, 2009 (29) | Germany | 2005-2007 | Evaluationsbericht 2005–2007  Ergebnisse des  Mammographie-Screening-Programms in Deutschland | CS | R | L2 | 54.3 | - | *Regional variables:*  Federal state |  |
| Evaluationsbericht, 2012 (30) | Germany | 2008-2009 | Evaluationsbericht 2008–2009  Ergebnisse des  Mammographie-Screening-Programms in Deutschland | CS | R | L2 | 53.7 | - | *Regional variables:*  Federal state |  |
| Evaluationsbericht, 2014a (13) | Germany | 2010 | Evaluationsbericht 2010  Ergebnisse des Mammographie-Screening-Programms in Deutschland | CS | R | L2 | 53.7 | - | *Regional variables:*  Federal state |  |
| Evaluationsbericht, 2014b (31) | Germany | 2011 | Evaluationsbericht 2011  Zusammenfassung der Ergebnisse des Mammographie-Screening- Programms in Deutschland | CS | R | L2 | 56 | 4,864,574 | *Regional variables:*  Federal state |  |
| Evaluationsbericht, 2015 (32) | Germany | 2012 | Jahresbericht Evaluation 2012 Deutsches Mammographie-Screening-Programm | CS | R | L2 | 56 | 4,881,399 | *Regional variables:*  Federal state |  |
| Evaluationsbericht, 2016a (33) | Germany | 2013 | Jahresbericht Evaluation 2013 Deutsches Mammographie-Screening-Programm | CS | R | L2 | 57 | 5,035,584 | *Regional variables:*  Federal state |  |
| Evaluationsbericht, 2016b (34) | Germany | 2014 | Jahresbericht Evaluation 2014 Deutsches Mammographie-Screening-Programm | CS | R | L2 | 54 | 5,267,337 | *Regional variables:*  Federal state |  |
| Evaluationsbericht, 2017 (35) | Germany | 2015 | Jahresbericht Evaluation 2015 Deutsches Mammographie-Screening-Programm | CS | R | L2 | 52 | 5,528,937 | *Regional variables:*  Federal state |  |
| Evaluationsbericht, 2018 (36) | Germany | 2016 | Jahresbericht Evaluation 2016 Deutsches Mammographie-Screening-Programm | CS | R | L2 | 51 | 5,495,899 | *Regional variables:*  Federal state |  |
| Evaluationsbericht, 2019 (37) | Germany | 2017 | Jahresbericht Evaluation 2017 Deutsches Mammographie-Screening-Programm | CS | R | L2 | 49 | 5,531,002 | *Regional variables:*  Federal state |  |
| Evaluationsbericht, 2020 (38) | Germany | 2018 | Jahresbericht Evaluation 2018 Deutsches Mammographie-Screening-Programm | CS | R | L2 | 50 | 5,708,761 | *Regional variables:*  Federal state |  |
| Evaluationsbericht, 2021 (39) | Germany | 2019 | Jahresbericht Evaluation 2019 Deutsches Mammographie-Screening-Programm | CS | R | L2 | 50 | 5,758,566 | *Regional variables:*  Federal state |  |
| Evaluationsbericht, 2022 (40) | Germany | 2020 | Jahresbericht Evaluation 2020 Deutsches Mammographie-Screening-Programm | CS | R | L2 | 49 | 5,445,380 | *Regional variables:*  Federal state |  |
| Evaluationsbericht, 2023 (14) | Germany | 2021 | Jahresbericht Evaluation 2021 Deutsches Mammographie-Screening-Programm | CS | R | L2 | 51 | 5,887,028 | *Regional variables:*  Federal state |  |
| Heinig, 2023 (15) | Germany | 2009-2018 | German mammography screening program: adherence, characteristics of (non-) participants and utilisation of non‑screening mammography—a longitudinal analysis | CH | C | N | 72 | 82,666 | Education | Preventive measures  Prevalence of comorbidities  Alcohol abuse  Tobacco abuse  Menopausal hormone therapy  Family history cancer |
| Kaucher, 2020 (49) | Westfalen-Lippe | 2013-2014 | Breast cancer incidence and mammography screening among resettlers in Germany | CS | S | N | 75.9 | 4,828 | Education  Migration background |  |
| Kuehnle, 2021 (48) | Hannover, Hameln, Hildesheim, and Stadthagen | 2012-2016 | First Prospective Cross-Sectional Study on the Impact of Immigration Background and Education in Early Detection of Breast Cancer | CS | R | N | 73 | 2,145 | Education  Migration background |  |
| Lemke, 2015 (45) | Dortmund | 2007-2012 | Small-area spatio-temporal analyses of participation rates in the mammography screening program in the city of Dortmund (NW Germany) | CH | R | L2 | - | 575,944 | *Regional variables:*  Unemployment rate (foreigner)  Unemployment rate (long-term)  Employment trend  Foreign residents’ rate  Unemployment rate (<25 aged)  Employment rate | *Regional variables:*  Living space per person  Birth rate  Female population rate  Social housing rate  Mortality rate  Youth quotient  Elderly quotient  Basic social welfare rate |
| Missinne, 2015 (20) | Germany | 2008-2009 | A cross-national comparative study on the influence of individual life course factors on mammography screening | CS | S | N | 48 | 896 | Education  Wealth | Childhood characteristics (ISCO breadwinner, presence of books, cultural health capital)  Availability of national screening program |
| Pokora, 2022 (21) | Rhine-Main-region | 2007- 2012 | Determinants of mammography screening participation–a cross-sectional analysis of the German population-based Gutenberg Health Study (GHS) | CH | S | N | 93.8 | 4,681 | Age  Education  Type of district  Income  Migration  Partnership cohabitation  Working  Health insurance | Children  Breastfeeding  Current smoker  Alcohol consumption  Diagnosed with cancer Mother diagnosed with breast cancer below age 50 |
| Starker, 2013 (43) | Germany | 2008-2011 | Participation in cancer screening programmes | CR | S | L2 | 71.3 | 1,174 | Age  Age-SES |  |
| Starker, 2017 (16) | Germany | 2014-2015 | Early detection of breast cancer: the utilisation of mammography in Germany | CR | S | L2 | 74.2 | 4,301 | Age  Age-education |  |
| Vogt, 2014 (41) | Germany | 2010-2011 | Examining regional variation in the use of cancer screening in Germany | CR | C | L1 | - | - | *Regional variables:*  Education  Income | *Regional variables:*  Voter turnout  Smokers’ prevalence  Travel time to mammography centre |
| Willems, 2018 (42) | Germany | 2006 | The education gradient in cancer screening participation: a consistent phenomenon across Europe? | CR | S | L1 | 46.4 | 237 | Education |  |

a (CR) cross-sectional (CH) cohort

b (S) self-reported (C) claims data (R) register based

c (L1) last-one year (L2) last-two years (N) never participated

**References**

47. Albert US, Kalder M, Schulte H, Klusendick M, Diener J, Schulz-Zehden B, et al. Das populationsbezogene Mammografie-Screening-Programm in Deutschland: Inanspruchnahme und erste Erfahrungen von Frauen in 10 Bundesländern. Das Gesundheitswesen. 2012;74(02):61-70.

46. Berens E-M, Stahl L, Yilmaz-Aslan Y, Sauzet O, Spallek J, Razum O. Participation in breast cancer screening among women of Turkish origin in Germany – a register-based study. BMC Women's Health. 2014;14(1):24.

44. Czwikla J, Urbschat I, Kieschke J, Schüssler F, Langner I, Hoffmann F. Assessing and Explaining Geographic Variations in Mammography Screening Participation and Breast Cancer Incidence. Frontiers in Oncology. 2019;9.

29. Evaluationsbericht 2005-2007. Ergebnisse des Mammographie-Screening-Programms in Deutschland. Berlin; 2009.

30. Evaluationsbericht 2008-2009. Ergebnisse des Mammographie-Screening-Programms in Deutschland. Berlin; 2012.

13. Evaluationsbericht 2010. Ergebnisse des Mammographie-Screening-Programms in Deutschland. Berlin; 2014.

31. Evaluationsbericht 2011. Zusammenfassung der Ergebnisse des Mammographie-Screening-Programms in Deutschland. Berlin; 2014.

32. Jahresbericht Evaluation 2012. Deutsches Mammographie-Screening-Programm. Berlin; 2015.

33. Jahresbericht Evaluation 2013. Deutsches Mammographie-Screening-Programm. Berlin; 2016.

34. Jahresbericht Evaluation 2014. Deutsches Mammographie-Screening-Programm. Berlin; 2016.

35. Jahresbericht Evaluation 2015. Deutsches Mammographie-Screening-Programm. Berlin; 2017.

36. Jahresbericht Evaluation 2016. Deutsches Mammographie-Screening-Programm. Berlin; 2018.

37. Jahresbericht Evaluation 2017. Deutsches Mammographie-Screening-Programm. Berlin; 2019.

38. Jahresbericht Evaluation 2018. Deutsches Mammographie-Screening-Programm. Berlin; 2020.

39. Jahresbericht Evaluation 2019. Deutsches Mammographie-Screening-Programm. Berlin; 2021.

40. Jahresbericht Evaluation 2020. Deutsches Mammographie-Screening-Programm. Berlin; 2022.

14. Jahresbericht Evaluation 2021. Deutsches Mammographie-Screening-Programm. Berlin; 2023.

15. Heinig M, Schäfer W, Langner I, Zeeb H, Haug U. German mammography screening program: adherence, characteristics of (non-)participants and utilization of non-screening mammography—a longitudinal analysis. BMC Public Health. 2023;23(1).

49. Kaucher S, Khil L, Kajüter H, Becher H, Reder M, Kolip P, et al. Breast cancer incidence and mammography screening among resettlers in Germany. BMC Public Health. 2020;20(1).

48. Kuehnle E, Siggelkow W, Luebbe K, Schrader I, Noeding K-H, Noeding S, et al. First Prospective Cross-Sectional Study on the Impact of Immigration Background and Education in Early Detection of Breast Cancer. Breast Care. 2020:1-7.

45. Lemke D, Berkemeyer S, Mattauch V, Heidinger O, Pebesma E, Hense H-W. Small-area spatio-temporal analyses of participation rates in the mammography screening program in the city of Dortmund (NW Germany). BMC Public Health. 2015;15(1).

20. Missinne S, Bracke P. A cross-national comparative study on the influence of individual life course factors on mammography screening. Health Policy. 2015;119(6):709-19.

21. Pokora RM, Büttner M, Schulz A, Schuster AK, Merzenich H, Teifke A, et al. Determinants of mammography screening participation–a cross-sectional analysis of the German population-based Gutenberg Health Study (GHS). PLOS ONE. 2022;17(10):e0275525.

43. Starker A, Saß AC. Inanspruchnahme von Krebsfrüherkennungsuntersuchungen. Bundesgesundheitsblatt - Gesundheitsforschung - Gesundheitsschutz. 2013;56(5-6):858-67.

16. Starker AK, Klaus; Kuhner, Ronny. Early detection of breast cancer: the utilization of mammography in Germany. Journal of Health Monitoring. 2017;2(4).

41. Vogt V, Siegel M, Sundmacher L. Examining regional variation in the use of cancer screening in Germany. Social Science & Medicine. 2014;110:74-80.

42. Willems B, Bracke P. The education gradient in cancer screening participation: a consistent phenomenon across Europe? International Journal of Public Health. 2018;63(1):93-103.
